# Exercise-Induced Bronchoconstriction in Children with Controlled Asthma Without Prominent Exercise-Related Symptoms: Prevalence and Associated Factors

**DOI:** 10.3390/children13091196

**Published:** 2026-09-04

**Authors:** Ercan Yılmaz, Nezihe Koker Ozer, Erdem Topal, Recep Günakın

**Affiliations:** 1 Department of Pediatric Allergy and Immunology, Faculty of Medicine, Inonu University, 44280 Malatya, Turkey; 2 Department of Pediatrics, Faculty of Medicine, Inonu University, 44280 Malatya, Turkey

**Keywords:** asthma, exercise-induced bronchoconstriction, atopic eczema, asthma control, children

## Abstract

**Highlights:**

**What are the main findings?**

• Exercise-induced bronchoconstriction was objectively detected in 35.8% of children with controlled asthma who did not report prominent exercise-related respiratory symptoms.
• A history of atopic eczema was associated with higher odds of EIB, although this finding should be interpreted cautiously and requires validation in larger cohorts.

**What are the implications of the main findings?**

• Adequate clinical asthma control, absence of prominent exercise-related symptoms, and preserved baseline lung function may not exclude EIB in children.
• A history of atopic eczema may help identify children with controlled asthma in whom objective assessment for otherwise unrecognized EIB should be considered.

**Abstract:**

**Background/Objectives**: Exercise-induced bronchoconstriction (EIB) may persist despite apparently adequate asthma control and may remain unrecognized in children without prominent exercise-related symptoms. This study aimed to determine the prevalence of objectively confirmed EIB in children with controlled asthma who did not report prominent exercise-related respiratory symptoms and to identify factors associated with EIB. **Methods**: This cross-sectional study included 67 children with controlled asthma who underwent standardized exercise challenge testing with serial spirometry. Demographic, environmental, allergic, laboratory, aeroallergen sensitization, and baseline pulmonary function characteristics were evaluated. Children were classified as EIB-positive or EIB-negative according to their post-exercise FEV_1_ response. Factors associated with EIB were assessed using univariate analyses and multivariable logistic regression. **Results**: EIB was identified in 24 of 67 children (35.8%). Baseline pulmonary function was generally preserved and did not differ significantly between EIB-positive and EIB-negative children. A history of atopic eczema was more frequent in the EIB-positive group (37.5% vs. 14.0%; *p* = 0.035). Allergic rhinitis, food allergy, total IgE, eosinophil percentage, and aeroallergen sensitization were not significantly associated with EIB. In multivariable logistic regression, a history of atopic eczema remained associated with EIB (adjusted odds ratio, 3.95; 95% CI, 1.08–14.41; *p* = 0.038), although the wide confidence interval indicates substantial uncertainty in the effect estimate. **Conclusions**: EIB was objectively detected in more than one-third of children with controlled asthma without prominent exercise-related symptoms despite preserved baseline pulmonary function. A history of atopic eczema may help identify children with persistent susceptibility to exercise-induced airway narrowing who might otherwise remain unrecognized.

## 1. Introduction

Exercise-induced bronchoconstriction (EIB) is characterized by transient narrowing of the lower airways during or, more commonly, following physical exercise and represents a frequent manifestation of airway hyperresponsiveness in children with asthma [1,2]. Although EIB can also occur in individuals without a diagnosis of asthma, its prevalence is substantially higher among asthmatic children. A systematic review and meta-analysis including 66 studies and 55,696 children and adolescents estimated the prevalence of objectively confirmed EIB at 9% in the general pediatric population and 46% among children and adolescents with asthma [3]. Because regular physical activity is essential for cardiovascular health, physical fitness, and normal childhood development, recognition of EIB is an important component of pediatric asthma management [1,2].

EIB is commonly considered a manifestation of inadequate asthma control and may reflect persistent airway hyperresponsiveness and underlying airway inflammation [1,4]. Consequently, EIB may be less readily suspected in children whose asthma appears clinically well controlled, particularly when they do not report prominent respiratory symptoms during physical activity. However, clinical asthma control and exercise-induced airway responses do not necessarily parallel each other, and previous studies have demonstrated that favorable asthma control assessments do not reliably exclude objectively confirmed EIB [5,6]. These observations suggest that adequate day-to-day symptom control may coexist with persistent exercise-induced airway hyperresponsiveness.

This discrepancy may be particularly relevant in children who do not report clear exercise-related respiratory complaints. Although cough, wheezing, dyspnea, chest tightness, and exercise intolerance may raise clinical suspicion of EIB, symptoms alone are insufficient to establish or exclude the diagnosis [1,2]. Children may fail to recognize mild exercise-related respiratory limitation, interpret respiratory discomfort as a normal consequence of exertion, or modify their level of physical activity in response to symptoms. Objective assessment is therefore important when EIB is clinically relevant, and standardized exercise challenge testing with serial FEV_1_ measurements provides direct evidence of exercise-induced airway narrowing [1]. Thus, the absence of prominent exercise-related symptoms may not reliably exclude EIB.

Most available studies of pediatric EIB have included heterogeneous asthma populations or have focused on children undergoing evaluation for exercise-related respiratory problems. Comparatively less is known about the burden of EIB among children whose asthma is clinically controlled and who do not describe prominent exercise-induced symptoms. This group is particularly relevant in routine practice because the clinical likelihood of EIB may appear low, while objective bronchoconstriction could remain undetected. Determining the prevalence of EIB in this apparently low-risk population may therefore clarify whether satisfactory clinical control and the absence of overt exercise-related complaints are sufficient to exclude exercise-induced airway dysfunction.

A further question is whether readily identifiable clinical or laboratory characteristics can identify children who remain susceptible to EIB despite otherwise controlled asthma. Eosinophilic airway inflammation, bronchial hyperresponsiveness, atopy, and airway obstruction have been investigated as potential determinants of EIB in asthmatic children [4]. Lee et al. demonstrated an association between eosinophilic inflammation and the severity of EIB in children with asthma [4]. In children with atopic asthma, Lex et al. found that exhaled nitric oxide was higher among those with EIB, whereas baseline lung function parameters did not reliably predict the exercise response [7]. Collectively, these findings suggest that resting pulmonary function and conventional clinical assessment may not fully identify children susceptible to exercise-induced airway narrowing.

The broader atopic phenotype may provide additional information. Childhood asthma frequently coexists with allergic rhinitis, food allergy, aeroallergen sensitization, and atopic eczema. Atopic eczema is particularly relevant because it commonly precedes respiratory allergic diseases within the concept of the atopic march [8,9]. Epidermal barrier dysfunction, IgE sensitization, shared genetic susceptibility, and type 2 immune responses have been implicated in the progression from cutaneous to respiratory allergic disease [8,9]. However, whether atopic eczema and other manifestations of atopy are associated with EIB specifically among children with controlled asthma and without prominent exercise-related symptoms remains insufficiently characterized.

Therefore, the present study aimed to determine the prevalence of objectively confirmed EIB in children with controlled asthma who did not report prominent exercise-related respiratory symptoms and to identify factors associated with EIB in this clinically stable population. We evaluated demographic, environmental, allergic, laboratory, aeroallergen sensitization, and baseline pulmonary function characteristics, with particular attention to components of the atopic phenotype, including histories of atopic eczema, allergic rhinitis, food allergy, and aeroallergen sensitization.

## 2. Materials and Methods

### 2.1. Study Design and Participants

This cross-sectional observational study included children and adolescents aged 8–18 years with physician-diagnosed asthma who were followed at a tertiary pediatric allergy and immunology center. The study was reported in accordance with the Strengthening the Reporting of Observational Studies in Epidemiology (STROBE) statement. Patients were eligible if their asthma was clinically controlled at enrollment, irrespective of maintenance asthma treatment, and they did not report prominent exercise-related respiratory symptoms during routine daily activities. The absence of such symptoms was determined by clinical interview and defined as no recurrent exercise-induced cough, wheezing, dyspnea, chest tightness, or activity limitation. Asthma diagnosis and control were assessed according to the Global Initiative for Asthma (GINA) recommendations [10], with the Asthma Control Test (ACT) or Childhood Asthma Control Test (c-ACT), as appropriate for age, used as an additional measure of symptom control. Patients with uncontrolled or partly controlled asthma were excluded from the study. Additional exclusion criteria included an acute respiratory tract infection within the preceding four weeks, an asthma exacerbation within the preceding three months, inability to perform acceptable spirometry or the exercise challenge test, contraindications to exercise testing, and chronic cardiopulmonary, neuromuscular, or systemic diseases that could independently affect exercise capacity or pulmonary function. A total of 67 children who fulfilled the eligibility criteria underwent standardized clinical assessment, baseline spirometry, and an exercise challenge test.

### 2.2. Clinical and Demographic Assessment

Demographic and clinical data were collected using a standardized case report form. Recorded variables included age, sex, height, weight, body mass index (BMI), duration of asthma, consanguinity, passive smoking exposure, breastfeeding for more than 6 months, presence of a pet in the household, and residence in a rural or urban area. Height and weight were measured using standard methods, and the corresponding z-scores were calculated according to age and sex. Anthropometric assessments were carried out in accordance with national reference data [11].

Personal allergic history included physician-diagnosed allergic rhinitis, atopic eczema, food allergy, and drug allergy. Family history of asthma, allergic rhinitis, and atopic eczema were also recorded. Asthma symptom control was evaluated using the ACT or c-ACT, as appropriate for age.

Particular attention was given to the presence of a previous history of atopic eczema because the study aimed not only to determine the frequency of EIB in clinically controlled asthma but also to identify clinical characteristics associated with persistent exercise-induced airway responsiveness.

### 2.3. Laboratory and Allergy Evaluation

Peripheral blood eosinophil percentage and total serum immunoglobulin E (IgE) levels were available for all participants, either from medical records or from measurements obtained as part of the clinical evaluation. Total IgE levels were expressed in IU/mL. Aeroallergen sensitization was assessed by skin prick testing and/or measurement of serum allergen-specific IgE. A skin prick test was considered positive when the mean wheal diameter was at least 3 mm greater than that of the negative control, whereas a serum allergen-specific IgE level ≥ 0.35 kUA/L was considered positive. Sensitization to pollen, house dust mite, mold, and cat dander was recorded separately, and polysensitization was defined as sensitization to two or more aeroallergen groups.

### 2.4. Respiratory Function Tests

Prior to the exercise provocation test, all participants underwent spirometry in accordance with the recommendations of the American Thoracic Society (ATS) and the European Respiratory Society (ERS) [12]. In the spirometric assessment, forced expiratory volume in the first second (FEV_1_), forced vital capacity (FVC), FEV_1_/FVC ratio, mid-expiratory flow rate (FEF25–75) and peak expiratory flow (PEF) values were recorded. At least three acceptable and reproducible maneuvers were obtained for each participant, and the highest values were used in the analysis. Results were expressed as percentages of the predicted values (% predicted) according to age, sex and height. In addition, Z-scores for the principal spirometric indices (FEV_1_, FVC, and FEV_1_/FVC) were calculated according to the Global Lung Function Initiative 2012 (GLI-2012) spirometry reference equations, incorporating age, sex, height, and ethnicity.

### 2.5. Exercise Provocation Test

The exercise provocation test was conducted in accordance with the guidelines on exercise-induced bronchoconstriction published by the American Thoracic Society [1]. All participants exercised on a treadmill, aiming to reach approximately 85–90% of their age-predicted maximum heart rate. The target exercise intensity was maintained for at least 6 min. Exercise testing was performed under standardized environmental conditions in an indoor laboratory, with an ambient temperature of 20–25 °C and relative humidity of <50%, in accordance with ATS recommendations [1]. To minimize potential pharmacological influences on airway responsiveness, participants were instructed to withhold medications before testing in accordance with ATS recommendations, including short-acting bronchodilators for at least 8 h, long-acting bronchodilators for at least 48 h, leukotriene receptor antagonists for at least 24 h, and other medications that could potentially influence airway responsiveness whenever clinically appropriate. Spirometric measurements were repeated at 5, 10, 15 and 30 min post-exercise. A decrease of 10% or more in FEV_1_ relative to the baseline value at any time point post-exercise was considered exercise-induced bronchoconstriction.

The change in FEV_1_ following exercise (ΔFEV_1_) was calculated as a percentage change relative to the baseline value. Symptoms such as dyspnea, cough, wheezing and chest tightness that arose during or after the test were recorded. Patients requiring bronchodilator treatment following the test were also recorded.

### 2.6. Study Outcomes

The primary outcome was the prevalence of objectively confirmed EIB among children with controlled asthma who did not report prominent exercise-related respiratory symptoms.

The secondary outcome was the identification of demographic, environmental, allergic, laboratory, sensitization, and baseline pulmonary function characteristics associated with EIB. Particular emphasis was placed on components of the atopic phenotype, including a personal history of atopic eczema, allergic rhinitis, food allergy, and aeroallergen sensitization.

### 2.7. Statistical Analysis

Statistical analysis was performed using SPSS Statistics (v.25.0; IBM Corp., Armonk, NY, USA). Normality was assessed using the Kolmogorov–Smirnov test. Continuous variables with non-normal distributions were presented as median and interquartile range (IQR), while categorical variables were expressed as numbers and percentages. Children were divided into EIB-positive and EIB-negative groups according to the exercise challenge test results. Continuous variables were compared between groups using the Mann–Whitney U test, while categorical variables were compared using the chi-square test or Fisher’s exact test, as appropriate. Given the limited number of EIB-positive events, a parsimonious multivariable model was used. Variables associated with EIB at *p* < 0.10 in the univariable analyses were considered for inclusion. Accordingly, history of atopic eczema, rural residence, and baseline FEV_1_ (% predicted) were entered simultaneously into the multivariable binary logistic regression model. No automated stepwise variable-selection procedure was used. The analysis was considered exploratory rather than predictive. EIB status was entered as the dependent variable, with EIB-negative coded as 0 and EIB-positive as 1. Adjusted odds ratios (aORs) with 95% confidence intervals (CIs) were calculated. A two-sided *p*-value < 0.05 was considered statistically significant.

### 2.8. Use of Generative Artificial Intelligence

Generative artificial intelligence (ChatGPT (GPT-5.6 Sol, OpenAI)) tools were used to assist with English-language and grammatical editing of the manuscript and with the preparation of graphical abstract. AI tools were not used for study conception or design, data collection or generation, statistical analysis, interpretation of the results, or development of the scientific discussion or conclusions. All scientific data presented in the graphical abstract are derived from the original study. All scientific content and AI-assisted outputs were critically reviewed and verified by the authors, who take full responsibility for the final content.

## 3. Results

### 3.1. Demographic, Clinical, and Laboratory Characteristics of the Study Population

A total of 67 children with controlled asthma were included; 44 (65.7%) were male, with a median age of 14 years (IQR, 4). The median ACT/c-ACT score was 22 (IQR, 3), consistent with the controlled asthma profile of the cohort. Allergic rhinitis was the most common accompanying allergic condition (62.7%), while 56.7% of participants were receiving active asthma treatment. Detailed demographic, anthropometric, clinical, environmental, laboratory, sensitization, and treatment characteristics are presented in Table 1.

### 3.2. Exercise Challenge Test Findings

Baseline spirometric parameters were generally preserved, and FEV_1_, FVC, and FEV_1_/FVC z-scores were within the normal range. Overall, EIB was objectively identified in 24 of 67 children (35.8%), with the highest frequency of positive responses occurring 15 min after exercise. Dyspnea was the most commonly reported symptom during testing, and 9 children (13.4%) required post-exercise bronchodilator treatment. Detailed baseline spirometric and exercise challenge findings are presented in Table 2.

### 3.3. Comparison of Children with and Without Exercise-Induced Bronchoconstriction

Children with and without EIB were generally comparable with respect to demographic, environmental, allergic, laboratory, sensitization, and baseline pulmonary function characteristics. A history of atopic eczema was more frequent in the EIB-positive group than in the EIB-negative group (37.5% vs. 14.0%; *p* = 0.035). Rural residence and baseline FEV_1_ (% predicted) also showed between-group differences with *p* values < 0.10, whereas spirometric z-scores did not differ significantly between groups. Active asthma treatment was similarly distributed between the groups. Detailed comparisons are presented in Table 3.

Based on the predefined univariable screening criterion (*p* < 0.10), history of atopic eczema, rural residence, and baseline FEV_1_ (% predicted) were entered simultaneously into the multivariable logistic regression model. In this exploratory model, a history of atopic eczema remained associated with EIB (aOR, 3.95; 95% CI, 1.08–14.41; *p* = 0.038). Rural residence showed a borderline association with EIB (aOR, 3.90; 95% CI, 0.93–16.39; *p* = 0.063). Similarly, higher baseline FEV_1_ (% predicted) showed a borderline association (aOR per 1% increase, 1.06; 95% CI, 1.00–1.12; *p* = 0.062). Given the limited number of EIB-positive events, these estimates should be interpreted as exploratory.

## 4. Discussion

The present study demonstrates that exercise-induced bronchoconstriction (EIB) may remain common even among children with clinically controlled asthma who do not report prominent exercise-related respiratory symptoms. More than one-third of our study population (35.8%) had objectively confirmed EIB during exercise challenge testing. Importantly, baseline pulmonary function was generally preserved and did not differ significantly between children with and without EIB. Among the clinical and allergic characteristics evaluated, the history of atopic eczema was more frequent in children with EIB and remained associated with EIB in the exploratory multivariable analysis. These findings suggest that satisfactory symptom control, absence of prominent exercise-related complaints, and preserved resting spirometry may not be sufficient to exclude exercise-induced airway hyperresponsiveness in children with asthma.

The prevalence of EIB observed in our population is clinically noteworthy given the deliberate inclusion of children with controlled asthma without prominent exercise-related symptoms. Our observed prevalence of 35.8% was somewhat lower than the pooled prevalence previously reported among children with asthma [3]. This difference may reflect variations in asthma severity, disease control, treatment, exercise protocols, and diagnostic thresholds. More importantly, our cohort represented a clinically selected group in whom EIB might not have been suspected on the basis of routine symptom assessment.

The discrepancy between perceived asthma control and objectively demonstrated EIB has previously been recognized. Prior studies have shown that exercise-induced airway responses may persist despite favorable asthma control assessments and that questionnaire-based assessment of exercise-related symptoms has limited concordance with objectively confirmed EIB [5,6,13]. These observations are consistent with our findings and are particularly relevant because our patients did not report prominent exercise-induced symptoms before testing. Children may underestimate mild respiratory symptoms, perceive breathlessness as a physiological consequence of exertion, or unconsciously restrict exercise intensity to avoid discomfort. Interestingly, despite the absence of prominent exercise-related complaints during routine daily activities, respiratory symptoms emerged during standardized exercise testing, with dyspnea being the most frequent (35.8%), whereas cough, wheezing, and chest tightness were less common. This discrepancy suggests that the absence of spontaneously reported symptoms does not necessarily indicate a symptom-free response to sufficiently intense exercise or exclude exercise-induced airway narrowing. Standardized exercise challenge may therefore reveal both respiratory symptoms and objective bronchoconstriction that remain unrecognized during usual activities.

Our findings regarding baseline pulmonary function further support this concept. Median baseline FEV_1_ was 99% predicted in children with EIB and 97% predicted in those without EIB, while FEV_1_/FVC and FEF25–75 were also comparable between groups. Thus, conventional resting spirometry did not discriminate between children with and without EIB. This is biologically plausible because EIB represents a dynamic response to increased ventilation rather than persistent resting airflow limitation. Previous studies have similarly suggested that baseline pulmonary function alone has limited ability to identify EIB in asthmatic children [7]. In children with preserved conventional spirometry, alternative physiological techniques may provide complementary information regarding peripheral airway dysfunction. Respiratory oscillometry, including forced oscillation and impulse oscillometry, assesses respiratory resistance and reactance during tidal breathing and may provide additional information on peripheral airway mechanics that is not apparent on conventional spirometry [14,15]. However, oscillometry should be considered complementary to, rather than a substitute for, standardized exercise challenge testing for the diagnosis of EIB, and the potential value of oscillometric measurements in identifying otherwise unrecognized exercise-related airway dysfunction warrants further investigation.

Atopic eczema was present in 37.5% of EIB-positive children compared with 14.0% of EIB-negative children. In the exploratory multivariable analysis, a history of atopic eczema remained associated with higher odds of EIB (aOR, 3.95; 95% CI, 1.08–14.41). However, the relatively small number of participants with atopic eczema and the wide confidence interval indicate considerable uncertainty regarding the magnitude of this association. Therefore, atopic eczema should not be regarded as an established predictor of EIB; rather, it may represent a potential clinical marker of an atopic phenotype characterized by increased susceptibility to exercise-induced airway narrowing. This hypothesis requires validation in larger prospective cohorts.

This observation has biological and clinical plausibility. Atopic eczema is often an early manifestation of the atopic march and is associated with subsequent development of respiratory allergic disease. Shared epithelial barrier abnormalities, genetic susceptibility, allergen sensitization, and type 2 inflammatory pathways may contribute to the progression from cutaneous disease to asthma and airway hyperresponsiveness [8,9,16,17]. Particularly relevant to our findings, Caffarelli et al. evaluated exercise responses in children with atopic eczema and detected EIB in 23% of affected children [18,19]. They also reported that clinical history was not sufficiently sensitive to identify children with a positive exercise challenge. Our study extends this observation to a different clinical setting: children with established but controlled asthma who do not have prominent exercise-related symptoms. In this population, a previous history of atopic eczema may therefore represent more than another allergic comorbidity and may indicate persistent airway susceptibility.

Allergic rhinitis was highly prevalent in our cohort and was numerically more frequent among children with EIB than among those without EIB (70.8% vs. 58.1%), although this difference was not statistically significant. This finding should be interpreted cautiously, as the relatively small sample size and high background prevalence of allergic rhinitis may have limited the ability to detect modest between-group differences. Furthermore, allergic rhinitis was recorded as a comorbidity without systematic assessment of its current severity, level of control, or treatment. These factors may influence airway inflammatory activity and could potentially modify the relationship between allergic rhinitis and EIB. Therefore, the absence of a statistically significant association in the present cohort should not be interpreted as evidence that allergic rhinitis is unrelated to EIB.

Other markers of the allergic phenotype, including food allergy, aeroallergen sensitization, polysensitization, total IgE, and peripheral blood eosinophil percentage, were also not significantly associated with EIB. Previous studies have linked EIB to eosinophilic inflammation; Lee et al., in a study of 268 children with asthma, found that eosinophilic inflammation was strongly related to EIB severity [4,20]. Differences from our findings may reflect the relatively small sample size, the controlled clinical status of our participants, treatment-related suppression of inflammation, or differences in the inflammatory markers evaluated. Moreover, peripheral blood eosinophilia may not accurately reflect contemporaneous airway inflammation.

Asthma treatment was heterogeneous in our cohort, with 56.7% of participants receiving active pharmacological treatment and 43.3% receiving no current treatment. Although the proportion receiving active treatment was similar between children with and without EIB, treatment status may nevertheless have influenced airway responsiveness. Medications with acute effects on bronchial responsiveness were withheld before exercise testing according to guideline recommendations; however, chronic controller therapy, particularly inhaled corticosteroid-containing regimens, may have modified underlying airway inflammation and the magnitude of the exercise response. Consequently, treatment heterogeneity may have contributed to variability in EIB expression and potentially attenuated associations with clinical or inflammatory characteristics. Because treatment dose, duration, and adherence were not systematically evaluated, the influence of chronic asthma therapy on these findings could not be fully determined.

The present study has several strengths. First, it focused on a clinically relevant but relatively understudied population: children with controlled asthma who did not report prominent exercise-related respiratory symptoms. Second, EIB was determined objectively using exercise challenge testing with serial post-exercise spirometry rather than inferred from symptoms. Third, we evaluated a broad range of demographic, environmental, allergic, laboratory, sensitization, and pulmonary function characteristics, allowing potential determinants of otherwise clinically unrecognized EIB to be explored. Nevertheless, several limitations should be acknowledged. The single-center design may limit the generalizability of our findings. In addition, the relatively small sample size and limited number of EIB-positive events restricted the number of covariates that could be included in the multivariable analysis and may have resulted in model instability and imprecise effect estimates, as reflected by the wide confidence intervals. Although a parsimonious three-variable model was therefore used, the regression findings should be considered exploratory and hypothesis-generating and require confirmation in larger cohorts. The cross-sectional design precludes causal inference, and a history of atopic eczema does not provide information regarding current disease activity or severity. Finally, airway inflammatory biomarkers such as fractional exhaled nitric oxide were not included, which might have provided further mechanistic insight.

## 5. Conclusions

Exercise-induced bronchoconstriction was objectively demonstrated in more than one-third of children with controlled asthma who did not report prominent exercise-related symptoms, despite generally preserved baseline pulmonary function. A history of atopic eczema was associated with EIB in this exploratory analysis and may represent a potential clinical marker of increased susceptibility to exercise-induced airway narrowing. These findings highlight a possible gap between apparent clinical asthma control and exercise-induced airway responsiveness and suggest that a history of atopic eczema may help identify children in whom residual EIB should be considered despite the absence of prominent exercise-related symptoms. Given the limited sample size and imprecision of the effect estimate, this association should be regarded as hypothesis-generating and requires validation in larger prospective cohorts before atopic history can be incorporated into strategies for selecting children for objective exercise testing.

## Figures and Tables

**Table 1:** Demographic, clinical and laboratory characteristics of the children with asthma (n = 67).

Category	Variable	n (%)
Demographics	Male sex	44 (65.7)
	Age, median (IQR), years	14 (4)
	Height z-score, median (IQR)	−0.2 (2.5)
	Weight z-score, median (IQR)	−0.12 (1.96)
	BMI z-score, median (IQR)	0.24 (2.33)
	Duration of asthma, median (IQR), years	5 (2)
	ACT/c-ACT, median (IQR)	22 (3)
	Parental consanguinity	9 (13.4)
	Passive smoking exposure	23 (34.3)
	Breastfeeding > 6 months	62 (92.5)
	Pet in the household	5 (7.5)
	Living in a rural area	12 (17.9)
Personal comorbidity	Allergic rhinitis	42 (62.7)
	History of atopic eczema	15 (22.4)
	History of drug allergy	0 (0)
	History of food allergy	5 (7.5)
Family history of atopy	Asthma	14 (20.9)
	Allergic rhinitis	28 (41.8)
	Atopic eczema	12 (17.9)
Laboratory	Total IgE, median (IQR), IU/mL	265 (207)
	Eosinophil %, median (IQR)	4.7 (4.4)
Aeroallergen sensitization	Pollen	47 (70.1)
Treatment	House dust mite	12 (17.9)
	Mold	4 (6)
	Cat dander	4 (6)
	Polysensitization	13 (19.4)
	Receiving active asthma treatment	38 (56.7)
	Inhaled corticosteroid	20 (29.9)
	Inhaled corticosteroid + long-acting β_2_-agonist	12 (17.9)
	Inhaled corticosteroid + montelukast	6 (9)
	No current asthma treatment	29 (43.3)

ACT: Asthma Control Test, c-ACT: Childhood Asthma Control Test.

**Table 2:** Exercise challenge test findings in children with asthma (n = 67).

Variables	n (%)
Baseline spirometry	
FEV_1_ (% predicted), median (IQR)	97.0 (13)
FEV_1_ z-score, median (IQR)	0.051 (0.89)
FVC (% predicted), median (IQR)	90 (14)
FVC z-score, median (IQR)	−0.08 (1.67)
FEV_1_/FVC (% predicted), median (IQR)	105 (8)
FEV_1_/FVC z-score, median (IQR)	0.44 (0.95)
FEF**_25–75_** (% predicted), median (IQR)	108 (27)
PEF (% predicted), median (IQR)	87 (26)
Exercise challenge test results	
EIB positivity at 5 min	6 (9)
EIB positivity at 10 min	6 (9)
EIB positivity at 15 min	11 (16.4)
EIB positivity at 30 min	7 (10.4)
Overall EIB positivity	24 (35.8%)
Fall in FEV_1_ from baseline, %, median (IQR), at 5 min	1 (5.9)
Fall in FEV_1_ from baseline, %, median (IQR), at 10 min	0 (3)
Fall in FEV_1_ from baseline, %, median (IQR), at 15 min	2 (6)
Fall in FEV_1_ from baseline, %, median (IQR), at 30 min	0 (4.1)
Exercise-related symptoms during testing	
Cough	9 (13.4)
Wheezing	3 (4.5)
Dyspnea	24 (35.8)
Chest tightness	2 (3)
Bronchodilator administration after test	9 (13.4)

**Note:** EIB positivity at individual post-exercise time points was not mutually exclusive; participants could meet the EIB criterion at more than one time point.

**Table 3:** Comparison of Clinical, Laboratory, and Baseline Pulmonary Function Characteristics Between Asthmatic Children with and Without Exercise-Induced Bronchoconstriction.

Variables	EIB (+) Group n = 24	EIB (−) Group n = 43	*p*-Value
Age, median (IQR), years	12 (5.5)	14 (4)	0.175
Male sex, n (%)	13 (54.2)	31 (72.1)	0.225
BMI z-score, median (IQR)	0.48 (1.34)	−0.17 (2.47)	0.875
Duration of asthma, median (IQR)	5 (4.5)	4 (2)	0.103
Personal history of allergic rhinitis, n (%)	17 (70.8)	25 (58.1)	0.43
Personal history of atopic eczema, n (%)	9 (37.5)	6 (14)	**0.035**
Personal history of food allergy, n (%)	3 (12.5)	2 (4.7)	0.341
Family history of asthma, n (%)	7 (29.2)	7 (16.3)	0.352
Family history of allergic rhinitis, n (%)	8 (33.3)	20 (46.5)	0.429
Family history of atopic eczema, n (%)	5 (20.8)	7 (16.3)	0.743
Breastfeeding > 6 months, n (%)	21 (87.5)	41 (95.3)	0.341
Passive smoking exposure, n (%)	8 (33.3)	15 (34.9)	1.0
Pet in house, n (%)	3 (12.5)	2 (4.7)	0.341
Living in a rural area, n (%)	7 (29.2)	5 (11.6)	0.09
Total IgE, median (IQR), IU/mL	265 (174.2)	265 (304)	0.339
Eosinophil %, median (IQR)	2.8 (3.58)	4.7 (7.7)	0.104
Pollen sensitization, n (%)	19 (79.2)	28 (65.1)	0.354
House dust mite sensitization, n (%)	2 (8.3)	10 (23.3)	0.188
Polysensitization, n (%)	2 (8.3)	11 (25.6)	0.114
Baseline FEV_1_ (% predicted), median (IQR)	99 (18)	97 (14)	0.082
Baseline FEV_1_ z-score, median (IQR)	−0.11 (1.91)	0.05 (0.90)	0.247
Baseline FEV_1_/FVC (% predicted), median (IQR)	107 (6)	105 (11)	0.642
Baseline FEV_1_/FVC, z-score, median (IQR)	0.60 (0.71)	0.44 (1.25)	0.315
Baseline FEF_25–75,_ (% predicted), median (IQR)	109 (20.75)	105 (41)	0.714
Receiving active asthma treatment, n (%)	14 (58.3)	24 (55.8)	1.0
Receiving ICS + LABA treatment, n (%)	5 (20.8)	7 (16.3)	0.728

BMI: body mass index; EIB: exercise-induced bronchoconstriction; FEF_25_–_75_: forced expiratory flow between 25% and 75% of forced vital capacity; FEV_1_: forced expiratory volume in 1 s; FVC: forced vital capacity; ICS: inhaled corticosteroid; IU: International Unit; IQR: interquartile range; LABA: long-acting β_2_-agonist.

## Data Availability

The data presented in this study are available on request from the corresponding author due to privacy.
